# Neutrophil elastase promotes macrophage cell adhesion and cytokine production through the integrin-Src kinases pathway

**DOI:** 10.1038/s41598-020-72667-3

**Published:** 2020-09-28

**Authors:** Karina Krotova, Nazli Khodayari, Regina Oshins, George Aslanidi, Mark L. Brantly

**Affiliations:** 1 Division of Pulmonary, Critical Care and Sleep Medicine, Department of Medicine, University of Florida, Gainesville, FL USA; 2 Hormel Institute, University of Minnesota, Austin, MN USA; 3 Division of Pulmonary, Critical Care and Sleep Medicine, University of Florida College of Medicine, 1600 SW Archer Rd Rm M454A, JHMHC PO Box 100225, Gainesville, FL 32610 USA

**Keywords:** Immunology, Inflammation, Innate immune cells, Inflammation

## Abstract

There are a number of respiratory diseases characterized by the presence of excess neutrophil elastase (NE) activity in tissues, including cystic fibrosis and chronic obstructive pulmonary disease (COPD). NE is considered a primary contributor to disease development, but the precise mechanism has yet to be fully determined. We hypothesized that NE alters the function of macrophages (Mɸ) which play a critical role in many physiological processes in healthy lungs. We demonstrate that monocyte-derived Mɸ exposed to NE releases active matrix metalloproteinases (MMPs), increase expression of pro-inflammatory cytokines TNFα, IL-1β, and IL-8, and reduce capacity to phagocytose bacteria. Changes in Mɸ function following NE treatment were accompanied by increased adhesion and cytoskeleton re-arrangement, indicating the possibility of integrin involvement. To support this observation, we demonstrate that NE induces phosphorylation of kinases from the Src kinase family, a hallmark of integrin signaling activation. Moreover, pretreatment of Mɸ with a specific Src kinase inhibitor, PP2 completely prevents NE-induced pro-inflammatory cytokine production. Taken together these findings indicate that NE participates in lung destruction not only through direct proteolytic degradation of matrix proteins, but also through activation of Mɸ inflammatory and proteolytic functions.

## Introduction

There is a growing interest in the role of neutrophil elastase (NE) in the pathology of a number of diseases associated with chronic inflammation, including lung and vascular diseases, obesity, and cancer^1–10^. NE is a serine protease found in abundance in neutrophils and stored in azurophil granules together with other proteins involved in anti-microbial defense. In response to infection or other inflammatory stimuli, neutrophils quickly infiltrate organs and release the contents of granules into the extracellular space. Released NE can reach µM concentrations locally to destroy pathogens^11–13^. However, it is quickly neutralized by protease inhibitors presented in extracellular and pericellular space to prevent destruction of host cells and extracellular matrix (ECM). Protease inhibitors such as the serpins, macroglobulins, and chelonianins are integral components of body fluids and typically effectively neutralize extracellularly-released NE, but in some pathological conditions unopposed protease activity occurs^2^. The most well-recognized example of such a situation is α1-antitrypsin (AAT) deficiency. AAT, a glycoprotein released by the liver into circulation, plays a major role in NE inactivation. Normal serum concentrations of AAT range from 20 to 53 µM; in AAT-deficiency (AATD), which can arise from several different mutations of the AAT gene, circulating AAT levels are typically fivefold less than normal^14–17^.

Individuals with AATD have an increased risk of developing pulmonary emphysema, a genetic form of chronic obstructive pulmonary disease (COPD), and the presence of unopposed NE is considered a major factor responsible for lung degradation. In other forms of emphysema, the role of NE is less obvious, but NE burden in the lungs correlates with the severity of disease^18,19^. Additional experimental data suggests that NE not only directly destroys tissues, but also triggers a prolonged pro-inflammatory response in lung resident cells, including airway epithelial cells, endothelial cells and Mɸ^20^. Alveolar Mɸ play a critical role in maintenance of lung homeostasis: at steady-state they produce low levels of inflammatory cytokines, maintain high phagocytic activity, and ability to suppress T cell activation^21,22^. However, lung diseases associated with chronic inflammation, such as COPD and cystic fibrosis, are characterized by alveolar Mɸ with altered phenotypes which support on-going inflammation in the lungs by producing inflammatory cytokines and participating in extracellular matrix remodeling^23^. Mice experiments highlight the importance of Mɸ in the development of NE-induced emphysema, as depletion of Mɸ in the lungs rescue mice from emphysema development^24^.

Given the importance of Mɸ in lung physiology, we estimated the direct effect NE has on Mɸ responses at concentrations typically found in the lungs of patients with moderate stage AATD and cystic fibrosis disease^25,26^. In this work we exposed human monocyte-derived Mɸ to 50–200 nM NE. We demonstrate that Mɸ exposure to NE leads to activation of several MMPs, increases expression of the pro-inflammatory cytokines IL-8, IL-1β, and TNFα, and reduces phagocytosis. In addition, we show that NE promotes integrin-mediated adhesion of Mɸ and Src kinase activation. Moreover, cytokine production by Mɸ in response to NE treatment is dependent on Src kinase activation since it can be inhibited by specific Src inhibitor, PP2.

Based on these data we propose a novel mechanism of NE-mediated activation of Mɸ innate immune responses through integrins and Src family kinases. Based on these findings inhibition of this pathway could be used as potential therapy to attenuate NE–induced inflammation in diseases characterized by the presence of free NE activity such as AATD.

## Results

### Profile of MMPs activated by NE treatment of Mɸ

**Figure 1:**
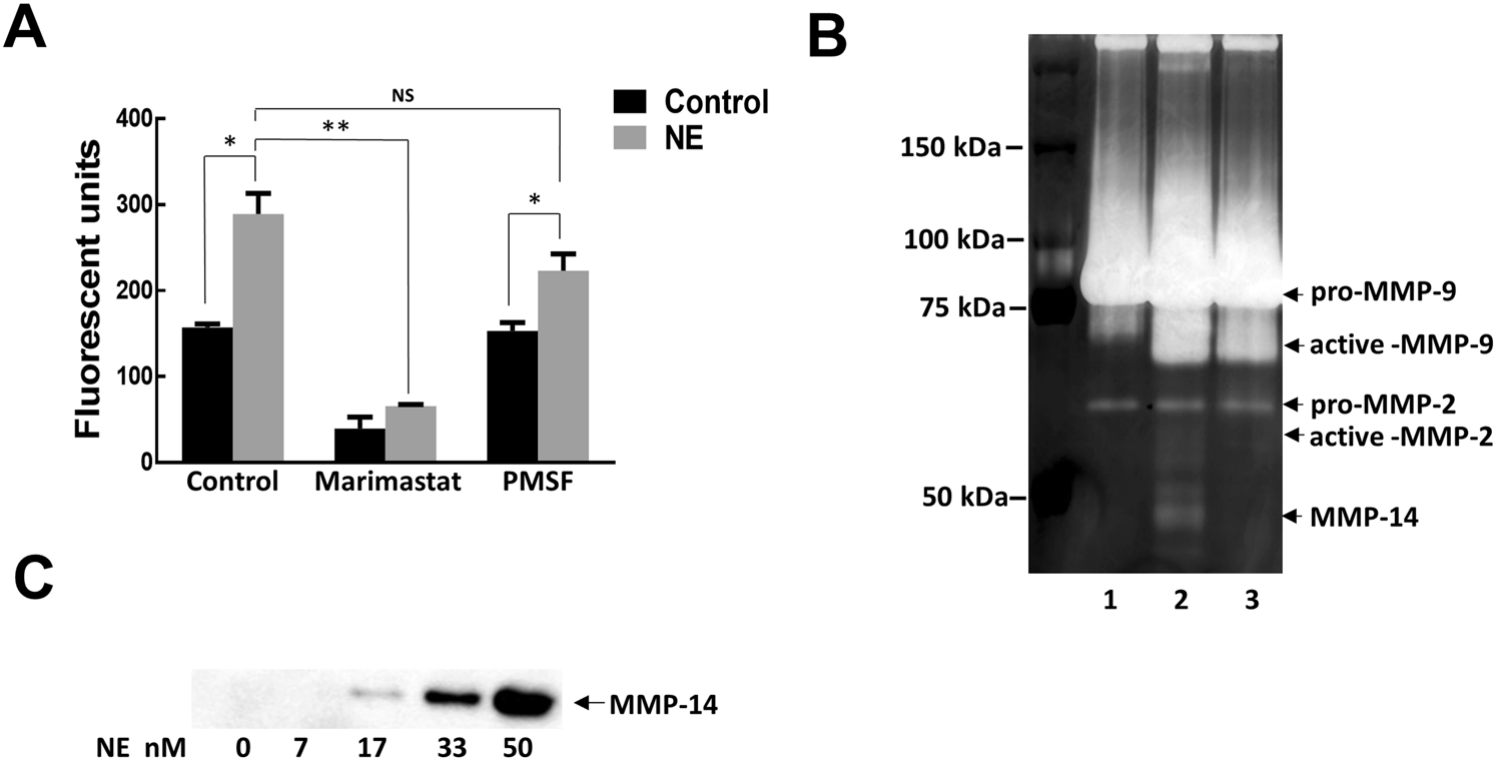
Increased Mɸ MMP activity after treatment with NE (50 nM). (**A**) Mɸ were treated with NE for 18 h. MMPs activity was measured in Mɸ conditioned media by measuring fluorescence released from the digested peptides. To confirm digestion of substrate was MMP-specific, the reaction was also performed in the presence of Marimastat (20 µM) or PMSF (2 mM). Data shown are representative of 3 independent experiments. *p < 0.05, **p < 0.01, *NS* not significant. (**B**) Gelatin zymography of Mɸ conditioned media. Lane 1—media from control cells. Lane 2—media from Mɸ treated with NE for 18 h. Lane 3—NE was added to conditioned media from control cells. Representative image of 4 independent experiments. (**C**) Immunoblot of MMP-14 present in conditioned media of macrophages after treatment with different concentrations of NE for 18 h. Representative image of 3 independent experiments with similar results.

To analyze the effect of NE on Mɸ-released MMP activity, conditioned media was collected after stimulation with NE (50 nM) for 18 h, and protease activity was measured by using MMP-specific fluorescent substrate. Exposure to NE significantly increased proteolytic activity in Mɸ conditioned media which was inhibited by marimastat (20 µM), a broad MMP-inhibitor, but not by PMSF (2 mM), a serine protease inhibitor (Fig. 1A). To determine which MMPs contributed to the elevated activity, we used gelatin zymography to detect proteases with gelatinase activity in both latent and active form. Conditioned media collected from control cells had a major band around 92 kDa and minor band around 70 kDa corresponding to latent forms of MMP-9 and MMP-2 (Fig. 1B, Lane 1). By contrast, the zymogram of conditioned media from Mɸ stimulated with NE revealed a number of new bands (Fig. 1B, Lane 2 vs Lane 1); under the pro-MMP-9 and pro-MMP-2 bands appeared new bands of active MMP-9 and active MMP-2; a ladder of several bands around 60–40 kDa were observed. All these bands represent metal-dependent enzymes such as the MMP family since incubation of the zymogram gel in the presence of EDTA completely blocked appearance of any bands (data not shown). Increased MMP activity in media was not due to transcriptional upregulation. There were no changes in mRNA expression of any of MMPs we studied in response to 50 nM NE (data not shown), though NE at higher concentrations (166–500 nM) can upregulate MMP-2 at the transcriptional level^27^. It is known that NE can directly cleave and activate the latent form of MMP-9^28^. It can also indirectly activate MMP-2, but only in the presence of cells which express MMP-14 on their surface^29^. Consistent with this fact we found that if to treat conditioned media collected from Mɸ with NE, it resulted in the appearance of the active form of MMP-9, indicating that MMP-9 is activated by NE directly. However, the band corresponding to active MMP-2 and bands around 60–40 kDa appeared only if NE was added to cells, not to conditioned media (Fig. 1B, Lane 3). We speculated that one of the bands around 60–40 kDa could be MMP-14. We found that treatment with NE causes MMP-14 to shed from Mɸ cell surfaces in a concentration dependent manner (Fig. 1C).

### NE upregulates inflammatory cytokine production in Mɸ

**Figure 2:**
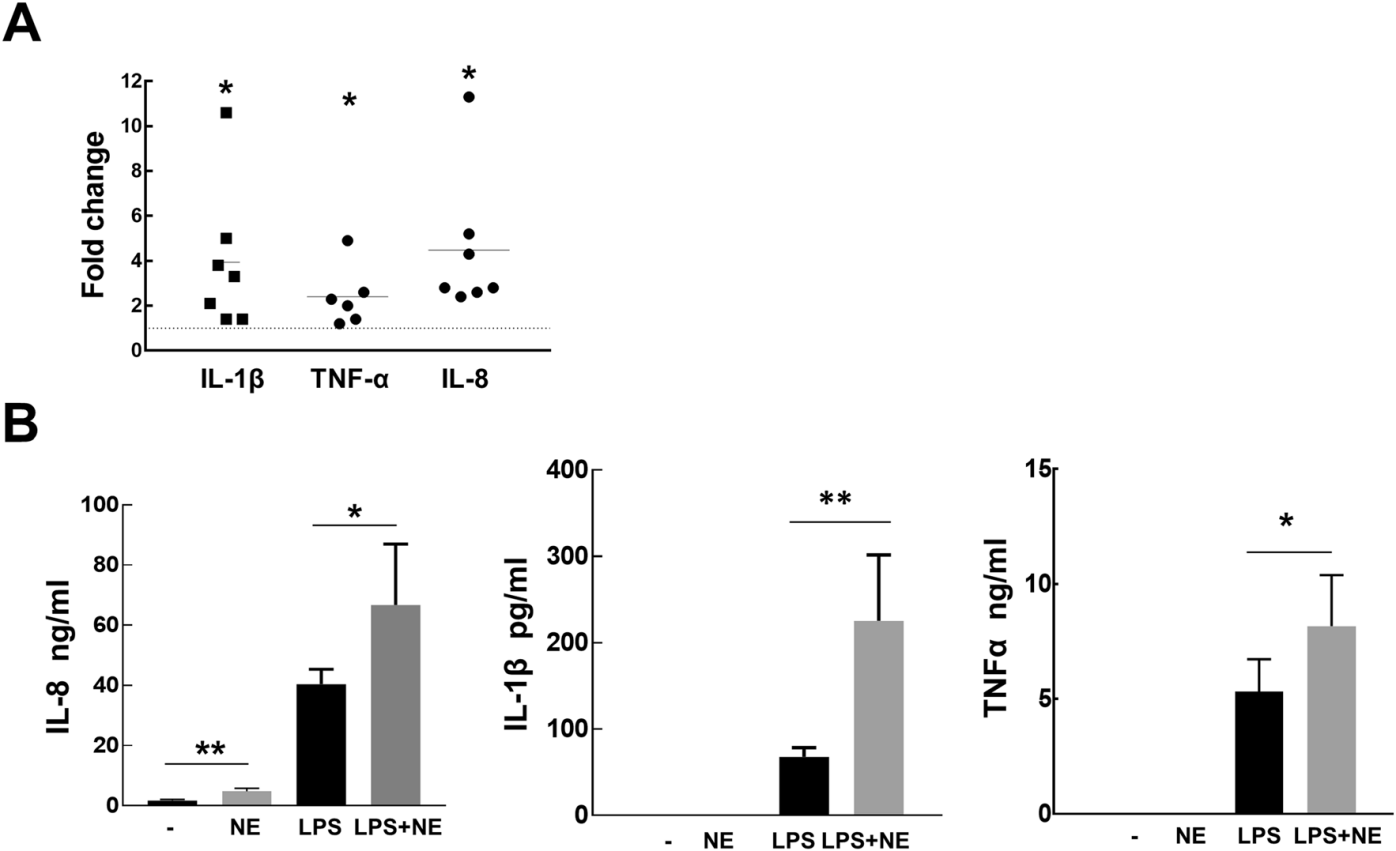
NE upregulates Mɸ cytokine levels. (**A**) Mɸ were treated with NE (50 nM) for 18 h. mRNA levels for IL-8, TNFα, and IL-1β were measured by real-time PCR. Each data point represents a single individual. Data are presented as fold of changes in NE treated Mɸ vs control cells where 1 corresponds to no changes. (**B**) Mɸ were treated with NE (50 nM), LPS (10 ng/ml), and LPS with NE for 18 h. Cytokine were measured in conditioned media. Graph represents cumulative data from at least five experiments using Mɸ from different individuals. t-test for NE treated vs control cells: *p < 0.05, **p < 0.01. Paired t-test for LPS plus NE treated vs LPS treated cells: *p < 0.05, **p < 0.01.

Next we analyzed changes in pro-inflammatory cytokines expressed by Mɸ in response to treatment with NE. Unstimulated Mɸ released low levels of IL-8, but not IL-1β, or TNFα. Exposure to NE (50 nM) resulted in increased mRNA levels of IL-8, IL-1β, and TNFα (Fig. 2A), but in conditioned media only IL-8 could be detected, for IL-1β and TNFα the sensitivity of ELISA was not sufficient (Fig. 2B). On average Mɸ treated with NE released 3 times more IL-8 (3.35 ± 1.13) compared to unstimulated Mɸ. To measure the changes induced by NE treatment on protein levels, Mɸ were co-stimulated with NE and a commonly used pro-inflammatory stimulus, LPS. In response to LPS treatment Mɸ released significant amounts of IL-8, IL-1β and TNFα, and NE was synergistic with LPS further increasing cytokine production (Fig. 2B).

### NE selectively cleaves some receptors, but not integrins, from the surface of Mɸ

**Figure 3:**
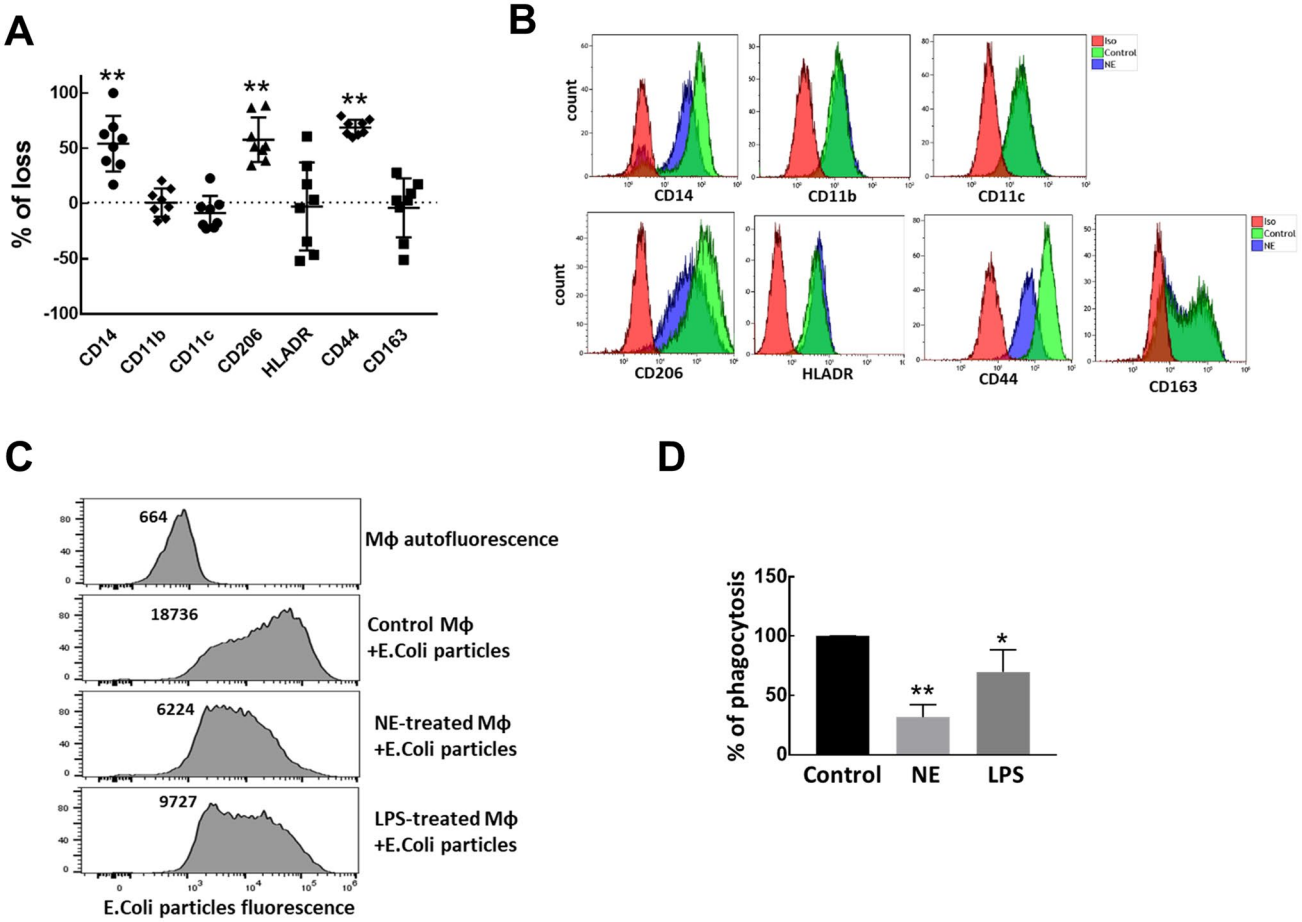
NE cleaves selected Mɸ surface receptors and reduces phagocytosis (**A**) Mɸ derived from 8 different individuals were treated with NE (200 nM) for 1hour and cell surface receptors were analyzed by flow cytometry. The data is presented as the percent of change in geometric mean of fluorescence intensity (MFI) for markers in NE treated cells relative to non-treated, control cells. t-test for NE treated vs control cells: **p < 0.01. (**B**) Representative images of changes in receptors after NE treatment for data summarized in (**A**). (**C**) Uptake of *E. coli*i particles conjugated with pHrodo red fluorescent dye measured by flow cytometry. Mɸ were untreated or pre-treated with LPS (10 ng/ml) or NE (50 nM) for 18 h before *E. coli* particles were added to cells for 1 h. Representative data from 3 independent experiments. (**D**) Summarized data of *E. coli* particles phagocytosis from 3 independent experiments. Phagocytosis by untreated macrophages was considered as 100%. *p < 0.05, **p < 0.01.

It has been shown that NE mediates many of its effects through cleavage of a number of cell surface molecules^1^. This is especially true when NE was used in µM concentrations^30,31^, however, the effect of lower nM concentrations could be different. We were interested in how NE treatment changes the levels of integrin subunits and other Mɸ cell surface markers. In our next experiments, we treated Mɸ with 200 nM NE. We analyzed the effect of NE on the cell surface markers we used to characterize PBMC-derived Mɸ: CD163, CD44, HLA-DR, CD14, CD206, CD11b, and CD11c. We found that NE treatment did not change the levels of integrin subunits CD11b and CD11c. In contrast, NE caused significant reduction on cell surface of CD14 (54.1% ± 25.1%), CD44 (68.8% ± 7.1%) and CD206 (57.8% ± 20.2%). The changes in HLA-DR and CD163 levels in response to NE were highly variable between Mɸ derived from different individuals and were not statistically significant (Fig. 3A,B). The decrease in the cell surface expression of receptors such as CD14 should affect Mɸ-mediated phagocytosis, one of the key functions in maintenance of lung homeostasis performed by Mɸ. Consistent with this fact the uptake of *E. coli* particles by NE-treated Mɸ was dramatically reduced, this reduction in phagocytosis was much stronger than in Mɸ treated with LPS (Fig. 3C,D).

### NE increases Mɸ spreading and adhesion

**Figure 4:**
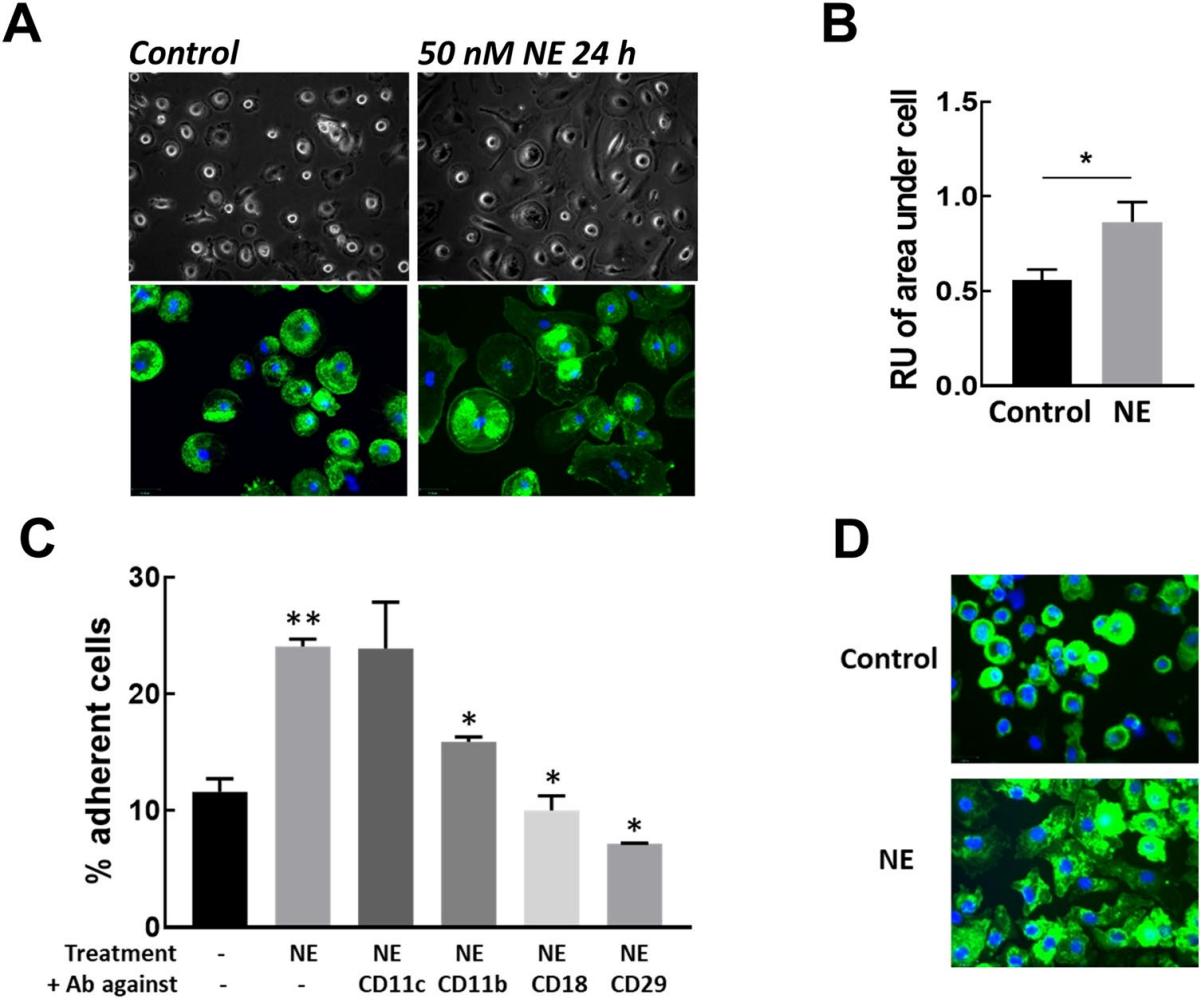
NE cause Mɸ spreading and increase adhesion. (**A**) Mɸ after incubation with NE (50 nM) for 24 h. Top panel—phase contrast image, bottom panel**—**after treatment with NE for 24 h, cells were fixed and F-actin was stained with FITC-phalloidin and nuclei were stained with DAPI. (**B**) Quantification of area under the cells which was expressed as a percentage of total view area. Data are representative of 3 independent experiments. *p < 0.05, control vs NE treated. (**C**) NE-induced Mɸ adhesion to fibronectin (Fbn) is integrin-dependent. The attachment of Mɸ to fibronectin coated plates in the presence of NE, with or without the pretreatment of Mɸ with integrin-specific antibodies. *p < 0.01 vs NE treated, **p < 0.01 vs Control (**D**) NE treatment induces dramatic changes in cytoskeleton. Mɸ were allowed to attach as in (**C**), then fixed and F-actin was stained with FITC-phalloidin and nuclei were stained with DAPI. All data are representatives at least three independent experiments.

NE cleaves a number of receptors and proteins from the Mɸ cell surface, but at the same time NE-treated Mɸ looked more spread out (Fig. 4A top panel). To quantify these changes, Mɸ were treated with NE for 24 h then area occupied by cells was calculated after F-actin was stained with FITC-phalloidin and nuclei were stained with DAPI (Fig. 4A bottom panel). We found significant changes in F-actin morphology and an increase in the area occupied by NE-treated Mɸ (Fig. 4B). The enhanced spreading of cells and changes in the cytoskeleton structure might indicate that NE treatment increases Mɸ adhesion. To test this hypothesis, we analyzed how NE affects the attachment of freshly seeded Mɸ by using an adhesion assay. Plating Mɸ on fibronectin-coated plates in the presence of NE dramatically increased the number of attached cells (from 11.6 ± 1.1% in control to 24.1 ± 0.6% in NE treated). Integrins are primary receptors which regulate cell adhesion to the extracellular matrix^32^, hence the increased Mɸ spreading might result from integrin activation. In agreement with this observation, we found the pre-incubation of Mɸ with integrin antibodies specific to CD11b, CD18, or CD29 prevented enhanced binding to fibronectin during NE treatment (Fig. 4C). We also showed that the plating of Mɸ in the presence of NE induced dramatic changes in cytoskeleton structure, another indicator of integrin activation (Fig. 4D).

### NE-induces Src phosphorylation and Src inhibitor prevents upregulation of cytokines

**Figure 5:**
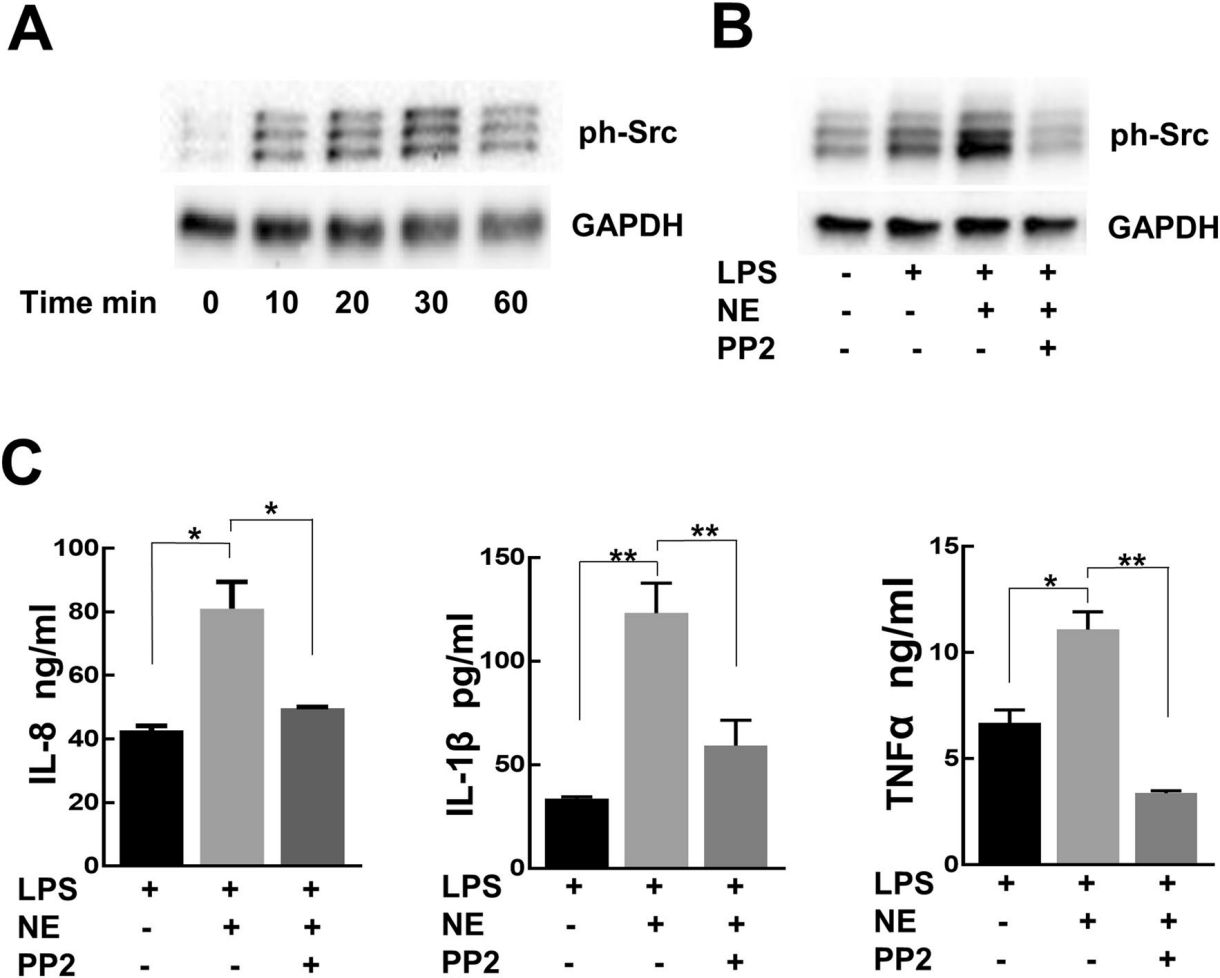
NE activates cytokine production through Src kinases pathway. (**A**) Time dependent phosphorylation of Src kinases in response to NE treatment. (**B**) Pretreatment of Mɸ with PP2 abolished Src phosphorylation by NE treatment. Representative images of 3 independent experiments. (**C**) The treatment with PP2 (2 µM) blocked NE-induced IL-8, IL-1β, and TNFα cytokine production. *p < 0.01, **p < 0.05.

Integrins participate in many cellular functions of immune cells through recruitment and activation of signaling proteins such as the members of Src non-receptor tyrosine kinase family^33–35^. We demonstrate that Mɸ exposed to NE have activation of the Src kinases signaling cascade. Activation of Src was determined by evaluation of phosphorylation of tyrosine residue 419^36^. NE induces phosphorylation of Src family members as early as 10 min after stimulation with NE, with maximum phosphorylation occurring within 30 min (Fig. 5A). Using non-isoform-specific antibodies we detected three bands in macrophage lysates, indicative that at least three members of Src family kinases respond to NE treatment by phosphorylation. To confirm the involvement of Src family kinases in NE signaling, we pre-treated Mɸ with the specific Src inhibitor, PP2 (2 µM), before stimulation with NE. PP2 completely abolished NE-induced phosphorylation of Src (Fig. 5B). NE-induced an increase in IL-8, IL-1β, and TNFα cytokine expression which was attenuated by pre-treatment of Mɸ with PP2 (Fig. 5C), indicating that Src kinases are involved in upregulation of these cytokines by NE.

## Discussion

The purpose of this study was to determine the role of NE in activating the inflammatory response of human Mɸ. These studies help define the role of Mɸ in tissue destruction during lung diseases characterized by the presence of unopposed NE activity, such as COPD and cystic fibrosis.

Concentrations of NE in the nM range were used in all experiments which corresponds to NE levels in the lungs of individuals with moderate COPD^25,37,38^. First, we showed that the incubation of human Mɸ with NE leads to a significant increase in MMP activity. This activity is comprised of several MMPs, including MMP-9, MMP-2, and MMP-14. We found that the effects of NE on MMP activity in Mɸ conditioned media were mediated through different mechanisms. MMP-9 and MMP-2 were released by Mɸ in the extracellular space in latent forms, and MMP-9 was activated by NE directly through direct proteolysis. MMP-2 was activated by NE indirectly, only in the presence of Mɸ, and transmembrane MMP-14 was shed from the Mɸ cell surface. While activation of MMP-9 and MMP-2 by NE was previously shown^28,29^, this study demonstrates the shedding of active MMP-14 from cell surfaces following exposure to NE. This finding is important particularly since soluble forms of MMP-14 with retained activity have been detected in asthma and other pathologic conditions but the mechanism of shedding was unknown^39,40^. Here we demonstrate that exposure of cells to free NE is a possible mechanism contributing to the presence of soluble forms of MMP-14. NE not only directly degrades ECM, but also activates several MMPs that further facilitate matrix degradation.

The presence of inflammatory cytokines in the lower respiratory tract is a hallmark of chronic inflammation presented by many lung diseases. We analyzed the effect of NE on the expression of pro-inflammatory cytokines secreted by Mɸ. Importantly, we found that 50 nM NE induced a several-fold increase in IL-8 mRNA and protein production by Mɸ. IL-8 is a potent neutrophil chemoattractant implicated in the pathogenesis of lung diseases such as COPD and cystic fibrosis^41,42^. A positive correlation between the levels of NE and IL-8 has been shown for COPD patients^37^, however the source of IL-8 production was not determined. A two-fold increase in IL-8 production under exposure to NE was also reported for airway epithelial cells^43–45^. In the lung, both epithelial cells and Mɸ can contribute to elevated expression of IL-8 in the presence of NE. In addition to stimulation of IL-8 production, the presence of NE significantly augments LPS-induced IL-1β and TNFα production. We observed an upregulation of cytokines at the transcriptional level consistent with a number of publications which demonstrate stimulatory effects of NE on cytokine levels both in mouse models and different types of human cells^43–48^. While the downregulation of cytokines through degradation by NE has been reported, these studies used NE doses much higher than used in the current study^31,38,49–51^. In summary, our data indicate that NE in concentrations found in COPD patients upregulates pro-inflammatory cytokines produced by human Mɸ that contribute to the chronic inflammatory environment in the lung^52,53^.

A number of publications point out TLR-4 receptor and NF-kB transcriptional factor as mediators involved in the stimulation of cytokine expression by NE^43,46^. In this study, we identified another pathway through which NE activate cytokine production. We show that in human Mɸ NE activates integrins and integrin-mediated intracellular signaling. We demonstrate that NE treatment dramatically increases Mɸ spreading and adhesion to fibronectin, and this effect can be abolished by antibodies specific to integrin’s subunits CD11b, CD29, and CD18.

The activation of Src kinases in response to NE stimulation further supports that NE targets integrins and integrin-mediated intracellular signaling. Our data complements previously identified signaling pathways triggered by NE. The Src kinase activation is an early response element in integrin signaling which leads to complex cell signaling cross-talk, including NF-kB activation^33^. It is interesting that the mechanism of NE-initiated inflammation via TLR-4 is associated with decreased TLR-4 surface expression^45^. In contrast, we found that though integrins are involved in NE signaling, their presence on Mɸ cell surfaces was not affected by NE treatment. At the same time, exposure to NE led to the significant reduction in cell surface expression of CD44, another adhesion molecule abundantly expressed on Mɸ. CD44 is a hyaluronic acid receptor that exhibits affinity to numerous factors, including collagen, fibronectin, chondroitin sulfate, osteopontin, and others^54^. Both integrins and CD44 are used by Mɸ for adhesion and migration and implicated in different pathologic conditions. Our data indicate that NE treatment changes the balance of adhesion receptors in favor of integrins, however the physiological significance of these findings is unclear and requires additional experiments. The activation of integrin signaling by NE may not be the only way to activate Mɸ. In addition to NE another serine protease, cathepsin G, activates integrin signaling and MIP-2 secretion in neutrophils^55^. The decrease in of receptors involved in phagocytosis, such as CD14, CD206, CD44, induced by NE could contribute to decrease of phagocytosis and bacterial clearance observed in COPD^56–58^. Accordingly, we showed that NE–treated Mɸ have reduced capacity for E. coli uptake.

Our study shows that NE increases integrin-mediated adhesion of Mɸ and modulates cytokine release from Mɸ through Src activation. This increase in cytokine expression can be abolished by Src kinase inhibitor PP2. Recently, activated Hck (hematopoietic cell kinase), a myeloid-specific Src family kinase has been implicated in lung–associated diseases^59,60^. Mice with a constitutively active Hck mutant develop areas of mild emphysema and fibrosis in their lungs^61^. Our work proposes the mechanism of Src kinase activation through the presence of free NE, however the link between free NE and Src kinase activation needs to be further study in vivo.

In conclusion NE can modulate multiple functions of Mɸ innate immune responses which may contribute to lung tissue destruction. Our data demonstrate a mechanism of NE action and provide further rationale for the use of NE inhibitors, as well as inhibitors of downstream pathways such as Src kinases, in diseases associated with NE-induced inflammation.

## Materials and methods

### Reagents

All cell culture reagents unless specified elsewhere, were from Life Technologies, (Carlsbad, CA, USA). All chemicals not specified are from Sigma-Aldrich (St. Louis, MO, USA). Human neutrophil elastase (NE) was from Athens Research & Technology (Athens, GA, USA). Collagen Type I from rat tail and Src inhibitor PP2 were obtained from EMD Millipore (Burlington, MA, USA). Human recombinant GM-CSF and M-CSF were from Peprotech (Rocky Hill, NJ, USA). pHrodo Red *E. coli* BioParticles Conjugate for phagocytosis was from Thermo Fisher Scientific.

### Cell culture

Primary peripheral blood mononuclear cells (PBMCs) were isolated from the blood of outpatient healthy volunteers. The University of Florida Institutional Review Board approved the study (UF IRB protocol 08-2007), which was conducted according to the principles of the Declaration of Helsinki. All participants signed written informed consent to participate in the study. Mɸ were differentiated from PBMC as described before^62^. Briefly, PBMCs were plated in serum-free RPMI and unattached lymphocytes were washed away after 1 h. Adherent monocytes were differentiated to Mɸ by culturing for 10 days in RPMI supplemented with 10% heat-inactivated FBS, 1 ng/ml GM-CSF, and 10 ng/ml M-CSF.

### MMP activity

MMP activity was assessed by a fluorogenic assay measuring 7-methoxycoumarin group (Mca) release from synthetic peptide Mca-PLGL-Dpa-AR-NH2 (RnD Systems, Minneapolis, MN, USA). After treatment with NE, media from Mɸ was collected, and aliquots of conditioned media mixed with MMP assay buffer containing 10 µM fluorogenic peptide. Changes in fluorescence were monitored hourly at 320/405 nm.

### Zymography

Media collected from unstimulated or NE-stimulated Mɸ were analyzed by gelatin zymography. Samples were mixed with 2 × Laemmli loading buffer without heating, and subjected to electrophoresis on 8% polyacrylamide gels containing gelatin (1 mg/ml). After gel electrophoresis was performed at 4 °C, gels were incubated in 2.5% (v/v) Triton-X100 for 30 min to recover enzymatic activity, then overnight in Tris (50 mM, pH 8.0), CaCl_2_ (5 mM), and ZnCl_2_ (1 µM) at 37 °C. At the end of incubation gels were stained with 0.125% Coomassie Blue. The presence of MMPs appears as transparent bands on blue background.

### Gene expression measured by real-time PCR (RT-PCR)

RT-PCR was conducted using Applied Biosystems TaqMan commercial primers on an ABI Prism 7500 fast detection system using standard protocols; 18S mRNA was used as an internal reference. Quantification of relative gene expression was performed using the comparative threshold cycle (C_T_) method^63^.

### Cytokine levels

IL-8 protein levels were measured in Mɸ conditioned media using a Biolegend ELISA kit (Biolegend, San-Diego, CA) with a range of 15.6–1000 pg/ml. Protein levels of IL-1β and TNFα were assessed using Luminex based kits (RnD systems, Minneapolis, MN) according to manufacturer’s recommendations. The standard curve for IL-1β ranged from 1.8 to 3997 pg/ml, and for TNFα from 1.3 to 2144 pg/ml. Samples with cytokine levels in the range of the standard curve were analyzed.

### Flow cytometry

After treatment, Mɸ were recovered by gentle scraping of cells from the plate using a cell lifter. Cell surface markers were analyzed by standard flow cytometry using a Beckman Gallios and post-analyzed using Kaluza software (Beckman Coulter, Indianapolis, IN). FITC-labeled anti-human CD14 and CD11b, PE-Cy7 labeled anti-human CD206, APC-labeled anti-human HLA-DR, and corresponding isotype controls were purchased from Beckman Coulter (Indianapolis, IN). APC-labeled anti-human CD163, PE-Cy7 labeled anti-human CD11c, FITC-labeled anti-human CD44 and corresponding isotype controls were from Biolegend (San Diego, CA). Before staining, Fc receptors were blocked with human BD Fc Block, and 7-AAD (BD Biosciences, San Jose, CA) was added 5 min before analysis to allow discrimination of dead cells.

### Cell spreading and adhesion assay

Mɸ spreading was determined by calculation of area under cells. Mɸ were treated with NE (50 nM) for 24 h, then fixed, stained with DAPI for nuclei and with FITC-phalloidin for F-actin, and visualized by fluorescence microscopy (Leica DMIRE2, magnification × 20). The quantification of cell area was assessed by image analysis software (Image J, 1.48 v, NIH). At least 100 cells in three random fields were analyzed for each sample. Results are presented as relative units (RU) of cell area.

For adhesion assays, wells in a 96-well plate were pre-coated with fibronectin (10 µg/ml in PBS) using 50 µl/well for 1hour at room temperature. Wells were then washed twice with PBS and free surface was blocked with Collagen I (50 µg/ml) for 1 h at room temperature. The choice of proper blocking agent is very important as Mɸ easily attaches to plastic. BSA, which is frequently used for blocking purposes, is not suitable as it binds Mɸ well. Hence, collagen I was chosen as a blocking agent for experiments with Mɸ as we found that Mɸ do not attach to it, and this observation was supported by independent studies^64,65^. Before experiments, Mɸ were cultured overnight in serum-free media. The next day cells were labeled with Calcein/AM (5 µg/ml) in HBSS/Ca^2+^/Mg^2+^ buffer for 20 min, then washed several times and detached by treating with accutase and gentle scraping. Cells were spun-down, suspended in HBSS/Ca^2+^/Mg^2+^, and treated with NE (50 nM) right before loading on to a coated plate. Some cells were pretreated with blocking antibodies to CD11b (Biolegend, clone ICRF44), CD18 (Biolegend, clone TS1/18), CD29 (Biolegend, clone P5D2), CD11c (Biolegend, clone Bu15) at a concentration of 50 µg/ml for 15 min on ice before adding the NE. Mɸ were plated on pre-coated 96-well plates, 100 µl per well at a concentration of 5 × 10^5^ cells/ml, and cells were left to adhere for 30 min at 37 °C in a CO_2_ incubator. At the end of incubation fluorescence was read at 480/520 nm, then wells were washed with PBS four times to remove unbound cells, and fluorescence was measured again. To calculate the percentage of attached cells the fluorescence intensity remained after wash (from attached cells) was divided by the total fluorescence before wash (from all cells).

### Western blot analysis

Mɸ lysates were separated by 7.5% or 10% SDS-PAGE and transferred to a nitrocellulose membrane. Membranes were blotted with specific antibodies to phospho-Src (Cell Signaling), MMP-14 (EMD Millipore), or GAPDH (Santa Cruz Biotechnology, Dallas, TX, USA).

### Statistics

All results are expressed as the mean ± S.E.M. Statistical analysis was performed using the two-tailed Student’s *t* test (GraphPad Prism 7.01 software; GraphPad Software, San Diego, CA, USA), and *p* < 0.05 was considered statistically significant. Data were plotted using GraphPad Prism 7.01.

## Supplementary information

Supplementary Information
