# Supplementary material for: Neutrophil elastase promotes macrophage cell adhesion and cytokine production through the integrin-Src kinases pathway
Source: Sci Rep. 2020 Sep 28;10:15874. doi: 10.1038/s41598-020-72667-3 (PMC7522083; doi:10.1038/s41598-020-72667-3)
Supplement: Supplementary file 1 — Supplementary Information [file 41598_2020_72667_MOESM1_ESM.docx]

Neutrophil Elastase promotes macrophage cell adhesion and cytokine production through the integrin-Src Kinases pathway

Karina Krotova^1,2^, Nazli Khodayari^1^, Regina Oshins^1^, George Aslanidi^2^, and Mark L. Brantly^1*^

^1^Division of Pulmonary, Critical Care and Sleep Medicine, Department of Medicine, University of Florida, Gainesville, Florida, United States

^2^Hormel Institute, University of Minnesota, Austin, Minnesota, United States

* Corresponding author. E-mail: [mbrantly@ufl.edu](mailto:mbrantly@ufl.edu)

**Supplementary materials: unprocessed full blots**

**
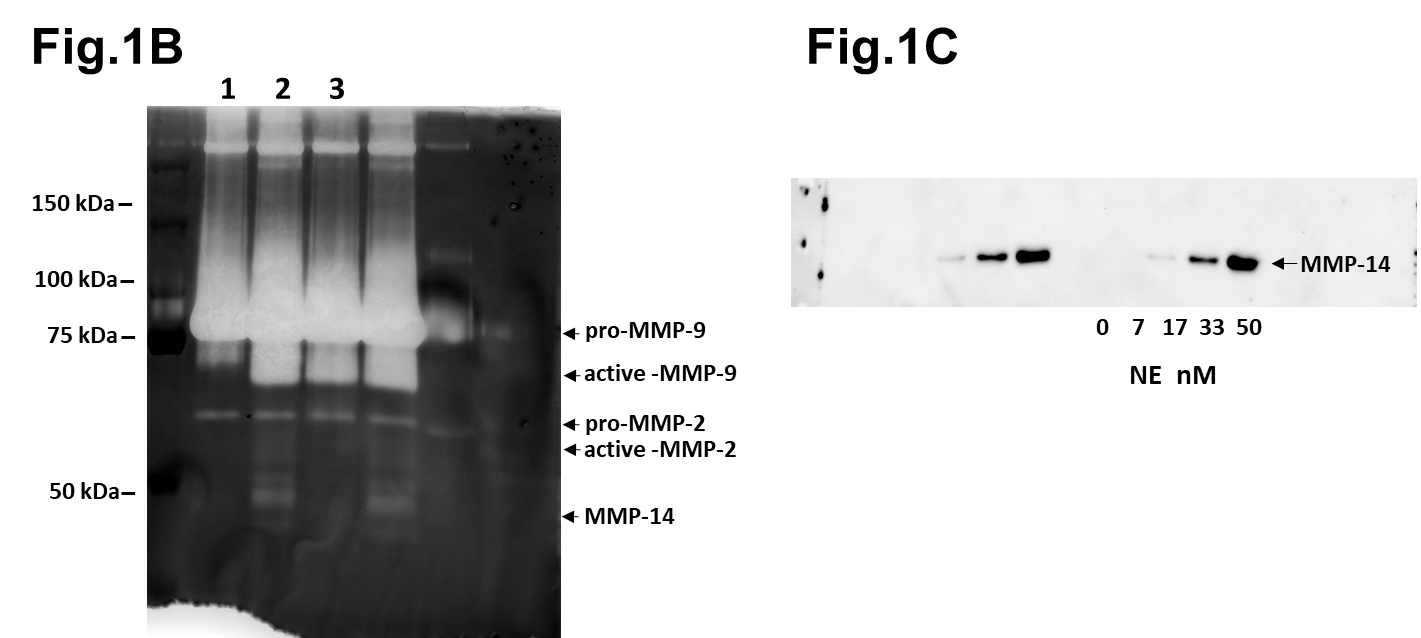
**

**
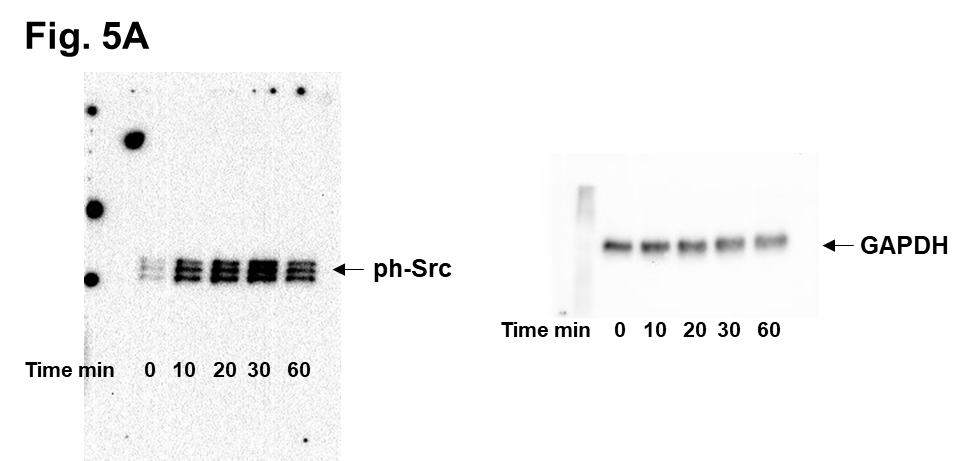
**

**
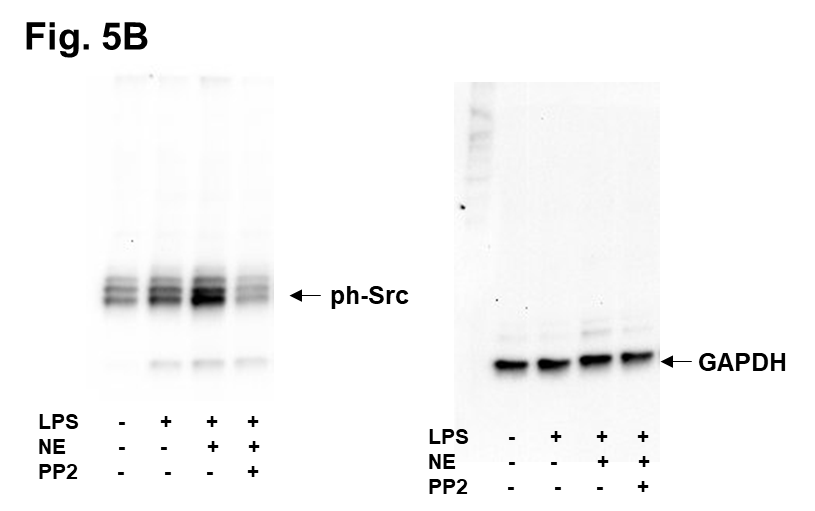
**
